# Protective coinfection: influenza reprograms myeloid cells to limit CD8 T cell–mediated malaria pathology

**DOI:** 10.21203/rs.3.rs-9404453/v1

**Published:** 2026-05-08

**Authors:** Jenna S. Reed, Ritika Nayan, Margot Deckers, Douglas H. Cornwall, Suzanne Ostrand-Rosenberg, Brian D. Evavold, Tracey J. Lamb

**Affiliations:** 1 Division of Microbiology and Immunology, Department of Pathology, University of Utah, Salt Lake City, UT, USA; 2 Department of Microbiology, Rega Institute for Medical Research, KU Leuven, University of Leuven, Leuven, Belgium.; 3 Huntsman Cancer Institute, University of Utah, Salt Lake City, UT, USA

**Keywords:** *Plasmodium*, lung injury, influenza, myeloid cells, immune suppression, CD8 T cells, Malaria, malaria-associated acute lung injury, co-infection, arginase, Plasmodium

## Abstract

Malaria-associated acute lung injury (MA-ALI) is a life-threatening complication of malaria driven by pathogenic CD8 T cell responses with no effective pharmaceutical interventions. Here, we show that co-infection with non-lethal influenza/A/HKx31 (X31) protects mice from malarial pulmonary vascular leak and death. X31 co-infection drove the expansion of Ly6C^+^ monocyte-derived alveolar macrophages, which inhibited pathogenic CD8 T cells in a contact-dependent manner. Moreover, *in vivo* blockade of monocytic myeloid cells with gemcitabine eliminated the protective phenotype. Protection occurred independently of parasite burden and did not require type I interferon signaling. Instead, co-infected pulmonary CD8 T cells exhibited broad transcriptional reprogramming and impaired inflammatory cytokine production. Our findings demonstrate that virus-induced myeloid cells suppress pulmonary CD8 T cells to prevent lung immunopathology in severe malaria. This work suggests that therapeutics that expand suppressive myeloid cells should be considered for adjunctive therapy for MA-ALI.

## Introduction

Malaria is a mosquito-borne parasitic disease that threatens over half the global population, causing an estimated 282 million cases and 610,000 deaths in 2024.^1^ Malaria-associated acute respiratory distress syndrome (MA-ARDS) and malaria-associated acute lung injury (MA-ALI)^2–5^ are severe manifestations of malaria that most commonly result from *Plasmodium vivax*^6^ or *P. knowlesi* ^7, 8^ infection, though they can also arise from infection with other *Plasmodium* species^9^. Currently, MA-ARDS / MA-ALI is treated by pairing mechanical ventilation with anti-malarial chemotherapies, but only 50% of patients receiving this care regimen survive^5, 10^. As such, novel strategies that work in conjunction with anti-parasitic drugs are needed to curtail pulmonary pathology and improve patient outcomes.

MA-ARDS / MA-ALI occurs when infected red blood cells (iRBCs) sequester and accumulate in alveolar capillaries, triggering inflammation and leukocyte recruitment^11^. Alveolar-capillary barrier dysfunction develops from tight-junction protein downregulation^12, 13^ and endothelial cytolysis by CD8 T cells^14^. This increases vascular permeability allowing protein-rich plasma to leak into the interstitial and alveolar spaces^15–18^. Co-infections can serve as a major complicating factor in clinical malaria leading to invasive bacterial disease with *Staphylococcus aureus* and non-typhoidal *Salmonella*^19^ as well as *Streptococcus pneumoniae*^20^. Interactions with viral infections range from suppression of innate responses to *Plasmodium* induced by concomitant HIV infection^21^ and association with hospitalization with malaria in acute EBV infections in young children^22^ to no reported exacerbation of malarial disease in the recent SARS CoVID2 pandemic^23^. Many pulmonary co-infections such as influenza are underreported and understudied in malaria endemic regions due to lack of diagnostic testing^24^. The impact of influenza co-infection on prognosis in malaria endemic areas is unclear despite the global prevalence of influenza.

We have used a mouse model of influenza-*Plasmodium* co-infection to determine that non-lethal influenza/A/HKx31 (X31) infection can protect mice from lethal malaria-associated pulmonary vascular leak. We identified a population of suppressive X31-induced pulmonary myeloid cells that were associated with dampening of inflammatory responses driven by otherwise pathogenic CD8 T cells during co-infection. These findings identify suppressive myeloid cells as an important therapeutic target for future treatment of MA-ARDS / MA-ALI.

## Results

### Influenza/A/HKx31 infection protects against *Pb*E-induced MA-ALI.

**Figure 1.**
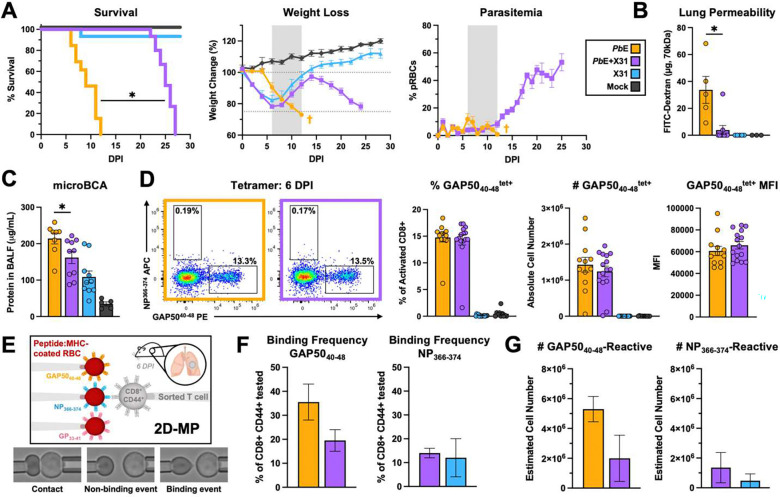
Coinfection with X31 protects against lethal *Pb*E infection. Co-infected mice lived significantly longer than *Pb*E singly-infected mice (p<0.0001; Kaplan-Meier Analysis with Mantel-Cox Test), despite no difference in early parasitemia (p=0.579; Mann-Whitney U of area under the curve (AUC) for 0–6 DPI) (n= 9–15 mice per group) **(A)**. *Pb*E+X31 mice exhibited less vascular leak than those singly-infected with *Pb*E as quantified by FITC dextran accumulation in the lung (p=0.003, Mann-Whitney U) **(B)** or protein accumulation in the BALF (p=0.0117, Mann-Whitney U) **(C)** at 6 DPI (n=5–10 mice per group). The frequency (p=0.6383), absolute cell number (p=0.5891), and median fluorescence intensity (MFI; p=0.2313) of CD44^hi^ CD62L^lo^ GAP50_40–48_^tet+^ CD8 T cells was not significantly different between co-infected and *Pb*E singly-infected mice at 6 DPI (Mann-Whitney U) (n=10–15 mice per group) **(D)**. Schematic of the 2D micropipette assay to quantify antigen-specific cells including both low and high affinity cells **(E)**. Similar numbers of CD44+ GAP50_40–48_ reactive and NP_366–374_ reactive CD8 T cells (both p=0.333, Mann-Whitney U) and were isolated from the lungs of *Pb*E singly-infected mice and *Pb*E+X31 co-infected mice at 6 DPI **(F)**. The number of NP_366–374_-reactive cells was not different between co-infected and X31 singly-infected mice (p=0.667; Mann-Whitney U) (n: 2 replicates, with 2 mice pooled per experiment; # cells tested: *Pb*E=48, *Pb*E+X31=51, X31=160) **(G)**. Data are represented as mean ± standard error of the mean (SEM).

Mice co-infected with *Plasmodium berghei NK65* Edinburgh clone (*Pb*E) and X31 survived longer than *Pb*E singly infected mice, ultimately dying of hyper-parasitemia (Figure 1A). This survival difference was not due to better parasite control, as parasitemia was the same between the *Pb*E and co-infected groups during the early infection. This protective phenotype was lung-specific, as X31 did not protect against death through breakdown of the blood brain barrier from *Plasmodium berghei* ANKA (*Pb*A) infection (Supplemental Figure 1A). Quantification of pulmonary vascular leak via intravenous (i.v.) injection of FITC-conjugated 70 kDa dextran (Figure 1B) or quantification of protein concentration in the bronchoalveolar lavage fluid (BALF) (Figure 1C) demonstrated that *Pb*E+X31 co-infected were protected from pulmonary vascular permeability. Thus, the pulmonary vasculature was more intact in co-infected mice.

Given that pulmonary vascular leak in *Pb*E is a CD8 T cell-mediated immunopathology, we tested whether X31 suppressed trafficking of *Pb*E-reactive CD8 T cells to the lung at 6 DPI when MA-ALI begins to develop. The frequency, absolute cell number, and median fluorescence intensity (MFI) of pulmonary glideosome-activated protein 50 tetramer-positive CD8 T cells (GAP50_40–48_; the dominant anti-*Plasmodium* epitope for CD8 T cells^25^) were similar between the *Pb*E+X31 co-infected and *Pb*E singly-infectced mice (Figure 1D). This was also mirrored by the number of total and activated (CD44^hi^ CD62L^lo)^ CD8 T cells (Supplemental Figure 1B). Very few activated CD8 T cells (<2%) stained with tetramer for the influenza nucleoprotein (NP_366–374_) in *Pb*E+X31 co-infected or X31 singly-infected mice at 6 DPI (Supplemental Figure 1C), expanding later in infection (Supplemental Figure 1D).

Tetramer staining only captures CD8 T cells that harbor T cell receptors (TCR) with high-affinity for antigen^26–29^. Using an established 2-dimensional micropipette adhesion frequency assay (2D-MP)^30–32^ (Figure 1E) to capture the total population of antigen-reactive CD8 T cells including those with T cell receptors (TCRs) with low affinity for antigen, we determined that 20–35% of activated pulmonary CD8 T cells bound specifically to GAP50_40–48_ and ~15% of the T cells bound NP_366–374_ in the *Pb*E singly infected and *Pb*E+X31co-infected mice (Figure 1F) . This data indicates that X31-reactive CD8 T cells in the lung at 6 DPI are contain a high proportion of lower affinity CD8 T cells. The total number of antigen-reactive (low and high affinity) cells in the *Pb*E+X31 co-infected mice displayed a trending decrease in pulmonary *Plasmodium*-reactive CD8 T cells compared to *Pb*E infection alone (Figure 1G). Together, these data demonstrate that X31 co-infection does not significantly alter the numbers of activated antigen-reactive CD8 T cells that traffic to the lung in response to *Pb*E infection.

### X31-induced protection only occurs during the innate phase of the immune response.

**Figure 2.**
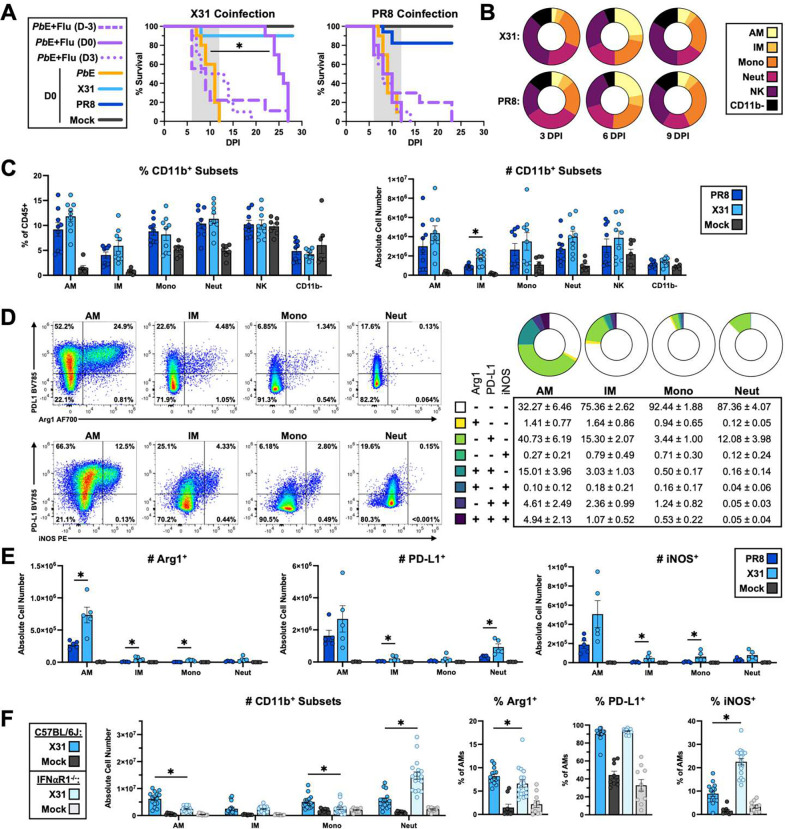
X31 induces a diverse myeloid response marked by high suppressive marker expression on pulmonary macrophages. X31-mediated survival of *Pb*E mice only occurs when mice were co-infected on the same day (Kaplan Meier Analysis with Mantel-Cox Test and Holm-Bonferroni correction for multiple comparisons D0 p<0.0001). There was no difference when mice were infected with X31 3 days before or 3 days after *Pb*E infection (n = 6–10 mice per group) **(A)**. Ly6C^+^ cells expanded to make up ~50% of the innate response at 6 DPI in both PR8 and X31 infected mice **(B)** where there was a robust accumulation of AMs, IMs, monos, and neutrophils (n = 5–17 mice per group) **(C)**. There was no difference in any of the Ly6C+ subsets between X31 and PR8 infected mice (all p>0.05) other than an increase in IMs in X31 singly-infected mice compared to PR8 singly-infected mice (absolute cell number p=0.033; Mann-Whitney U). Representative flow plots of suppressive marker expression staining (left) and the relative frequencies of CD11b^+^ subsets that express one, two, or all three suppressive markers (right; mean±SEM) in X31 infected mice at 6 DPI are shown **(D)**. AMs express a variety of suppressive molecules during X31 infection, with ~68% of cells expressing at least one molecule, ~25% expressing 2 or more suppressive molecules (n=3–5 mice per group). Arg1 was significantly higher in all Ly6C^+^ subsets (all p=0.031) and trending towards significance for neutrophils in X31-infected mice compared to PR8-infected mice (p=0.056) **(E)**. PD-L1 was higher in IMs and Neuts while iNOS was higher in IMs and monocytes (all p=0.031) and trending towards significance in AMs and Neuts (both p=0.062) (Mann-Whitney U: PR8 vs. X31). X31-infected IFNαR1^−/−^ mice had decreased AM and monocyte populations and an increased frequency and absolute cell number of neutrophils compared to X31-infected C57BL/6J mice (all p<0.001; Mann-Whitney U) (n= 9–15 mice per group) **(F)**. Both the frequency (p=0.022) and number (p<0.01) of Arg1^+^ AMs was decreased in the absence of type 1 interferon signaling. The number of PD-L1+ AMs was also decreased in IFNαR1^−/−^ mice (P<0.001). The % of iNOS+ AMs was significantly increased in IFNαR1^−/−^ mice (p<0.001) while the number of iNOS^+^ cells was not significantly different (p=0.741). Data are represented as mean ± SEM. AM: Alveolar Macrophage; IM Interstitial Macrophage; Mono: monocyte; Neut: neutrophil; NK: Natural Killer Cell

We observed limited protection when infecting mice with X31 3 days after *Pb*E infection suggesting the target protective response is short-lived (Figure 2A). Co-infection with a non-lethal dose of the related strain influenza/A/PR8/8/34 (PR8) did not protect mice from death by MA-ALI (Figure 2A). X31 and PR8 share identical genes for internal proteins,^33^ differing only in surface proteins (hemagglutinin/HA; neuraminidase/NA), yet they exhibit differences in lethality due to early proinflammatory cytokine production^34^. This supports the hypothesis that the protective effects of X31 was due to a difference in the induction of the innate immune response.

We quantified pulmonary innate immune cell populations expanded by X31 and PR8 infection (Supplementary Figure 2B) and identified a population of immature monocyte-derived Ly6C^+^ alveolar macrophages (AM; CD11b^+^ Ly6C^+^ CD11c^+^ F4/80^+^ CD64^+^) that re-populate the alveolar niche following influenza-mediated depletion of tissue-resident alveolar macrophages (rAM)^35^. At 6 DPI, we found that Ly6C^+^ AMs formed a large portion of expanded cells in both X31 and PR8 infections (Figures 2B and 2C). Most X31-induced AMs (~68%) expressed one or more of the suppressive molecules arginase 1 (Arg1), inducible nitric oxide synthase (iNOS) and programmed cell death ligand 1 (PD-L1) (Figure 2D). These molecules have been demonstrated to suppress anti-tumor CD8 T cell responses^36, 37^. These suppressive molecules were not as highly expressed in other CD11b^+^ immune cell compartments (interstitial macrophages/IM: CD11b^+^ Ly6C^+^ CD11c^−^ MHCII^+^ F4/80^+^ CD64^+^; monocytes/mono: CD11b^+^ Ly6C^+^ CD11c^−^ MHC-II^−^; or neutrophils/neut: CD11b^+^ Ly6G^+^). Only a small number of PR8-induced AMs at 6 DPI expressed suppressive molecules (Figure 2E). This correlates with the inability of PR8 infection to protect against *Pb*E-induced CD8 T cell-mediated MA-ALI. The frequency of pulmonary Arg1^+^ and PD-L1^+^ AMs was highest at 6 DPI during X31 infection (Supplemental Figure 3A–B) coinciding with the onset of MA-ALI. Expansion of this cell population was dependent on live X31 virions, as neither infection with a heat-killed X31 (Supplemental Figure 3C–E), nor a high dose of PR8-based live-attenuated vaccine (LAIV) (Supplemental Figure 3F–G), induced significant macrophage expansion nor the expression of suppressive molecules.

Type I interferons (IFN) are an essential component of the innate anti-viral response and elevated type I IFN levels are associated with more lethal influenza infection^38, 39^. Monocyte and macrophage responses during viral infection can be impacted by type I IFNs, though their effects are highly context dependent^40, 41^. Infection of IFN-α-receptor-1-deficient (IFNαR1^−/−^) mice led to a drastic reduction in the number of AMs (Figure 2F) and a lower proportion of IFNαR1^−/−^ AMs expressing Arg1, while there was no change in PD-L1 frequency and an increase in the percent expressing iNOS (Figure 2F). Together, these results indicate that type 1 interferon signaling is crucial for mobilizing the AM response during X31 infection, but it is not the sole driver of suppressive molecule expression in these cells. PR8 infection induces more type I interferon than X31 infection,^39^ yet PR8 induces similar total numbers of myeloid subsets (Figure 2E) but fewer producing suppressive molecules (Figure 2E). This may be why PR8 provides no protection against *Pb*E-induced MA-ALI. Together, these results demonstrate that type I interferon is not required for driving X31-induced protection against MA-ALI.

### X31-induced pulmonary macrophages suppress CD8 T cells *in vitro*.

**Figure 3.**
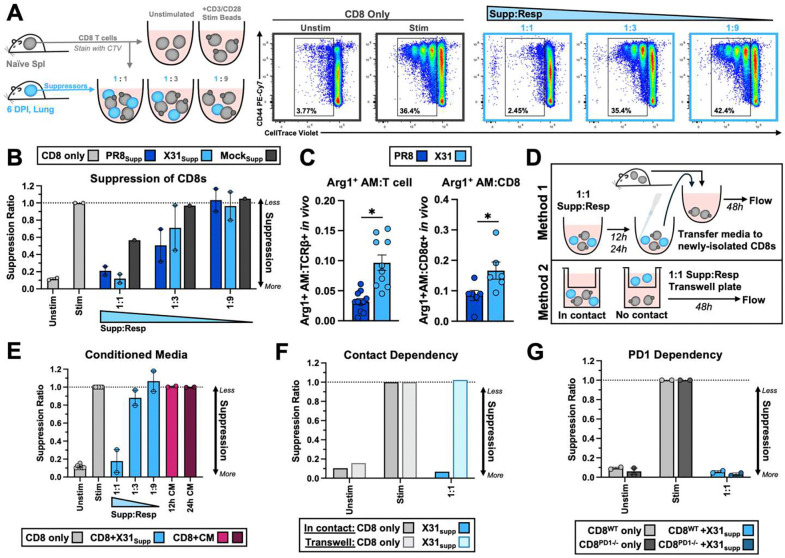
Monocytic cells derived from X31-infected lungs suppress CD8 T cell proliferation *in vitro* in a contact-dependent manner. Pulmonary Ly6C^+^ cells (AM, IM, Mono) from the lungs of X31-infected and PR-8 infected mice at 6 DPI were cultured with naïve CD8 T cells labelled with CTV. Right: Representative flow plots demonstrating proliferation (x-axis: CTV) and activation (y-axis: CD44) of CD8 T cells in each group. CD8 T cells cultured at a 1:1 ratio of X31-induced Ly6C^+^ cells fail to proliferate and activate **(A)**. Both PR8-induced and X31-induced pulmonary Ly6C+ cells suppressed CD8 T cell proliferation at the 1:1 ratio (PR8 p=0.742, X31 p>0.999; Kruskal-Wallis: 1:1 vs. Unstim) (n= two experimental repeats with biological replicates) **(B)**. Based on absolute cell numbers from flow cytometry data at 6 DPI, the ratio of Arg1^+^ AMs in the lung was divided by the number of total T cells or CD8 T cells to infer the ratio of suppressors to responders that occurs *in vivo* (n: PR8=5 mice, X31=6 mice). X31 infection induces a significantly higher ratio of Arg1^+^ AMs to T cells and CD8 T cells than PR8 infection (Arg1^+^ AM:T cell p=0.0001, Arg1^+^ AM:CD8 p=0.0087; Mann-Whitney U) **(C)**. To determine if the mechanism of suppression is contact dependent X31-induced Ly6C^+^ subsets were cocultured at a 1:1 ratio with stimulated CD8 T cells to condition media with monocyte-secreted suppressive soluble factors for 12 or 24 hours **(D)** or in a transwell plate. Neither conditioned media (CM) (E) nor pulmonary Ly6C^+^ cells plated in transwells (**F**) were able to suppress CD8 T cell proliferation *in vitro* (CM n = 2 biological replicates; transwell n= 1 biological replicate). X31-induced pulmonary Ly6C^+^ cells were able to suppress PD1−/− responder CD8 T cells to the same extent as intact responder CD8 T cells (p=0.333; Mann-Whitney U), signifying that PD1-PD-L1 interactions are not required for suppression *in vitro* (n=2 biological replicates) **(G)**. Data are represented as mean ± SEM.

We quantified the suppressive capacity of X31-induced pulmonary monocytic populations (AMs, IMs, monos) by co-culturing them with activated CellTrace Violet (CTV)-labelled CD8 T cells (Figure 3A). When co-cultured with higher ratios (1:1) of X31-induced monocytic suppressor cells, CD8 T cell proliferation was suppressed when compared to stimulated CD8-only positive control wells (Figure 3B). Equivalent suppressor populations isolated from PR8-infected lungs at 6 DPI had similar suppressive capacity (Figure 3B). However, based on our flow cytometry data, PR8 infection produces fewer Arg1^+^ AMs in proportion to CD8 T cells *in vivo*, making them less likely to suppress CD8 T cells in the context of PR9 infection (Figure 3C).

Expression of suppressive markers by myeloid cells is often tied to the inflammatory microenvironment^42^ and they can play redundant roles in the suppressive function of these cells^43^. Suppressive Ly6C^+^ cells may act through soluble factors such as Arg1 and iNOS, or through contact dependent molecules such as PD-L1. To test suppression was contact-dependent, we performed two modified suppression assays (Figure 3D). We generated conditioned media (CM) by co-culturing X31-induced pulmonary Ly6C+ monocytes/macrophages with stimulated CD8 T cells, allowing any suppressive soluble factors to accumulate (Figure 3D, **top**). Freshly-isolated CD8 T cells were cultured with stimulation beads in the presence of CM but no suppressive effect on CD8 T cell proliferation was observed compared to when CD8 T cells were stimulated with the pulmonary Ly6C^+^ cells (Figure 3E). Pulmonary Ly6C^+^ cells were also plated in transwell plates, preventing them from coming into contact with the responder cells (Figure 3D, **bottom**) but were unable to inhibit CD8 T cell proliferation without cell contact (Figure 3F). The finding that the suppression mechanism is contact-dependent is supported by data showing that inhibitors of Arg1 (norNOHA) or iNOS (L-NAME, to ablate NO generation) did not block the suppressive ability of pulmonary Ly6C^+^ cells (Supplementary Figure 4A, B).

Although most X31-induced AMs expressed PD-L1, X31-induced pulmonary Ly6C^+^cells retained the ability to inhibit PD1^−/−^ CD8 T cell proliferation *in vitro* (Figure 3G), indicating that PD-L1–PD-1 signaling is not required for suppression. We also found that they were able to suppress intact CD8 T cell proliferation in the presence of αPD-L1/L2 blocking antibodies (Supplementary Figure 4C). These results indicate that X31-induced monocytes/macrophages express additional surface molecules that mediated CD8 T cell suppression and that the suppressive mechanism is likely different than that of monocytic suppressor cells found in other models such as tumors.

### X31-induced Ly6C^+^ monocytes/macrophages are required for protection against *Pb*E infection.

**Figure 4.**
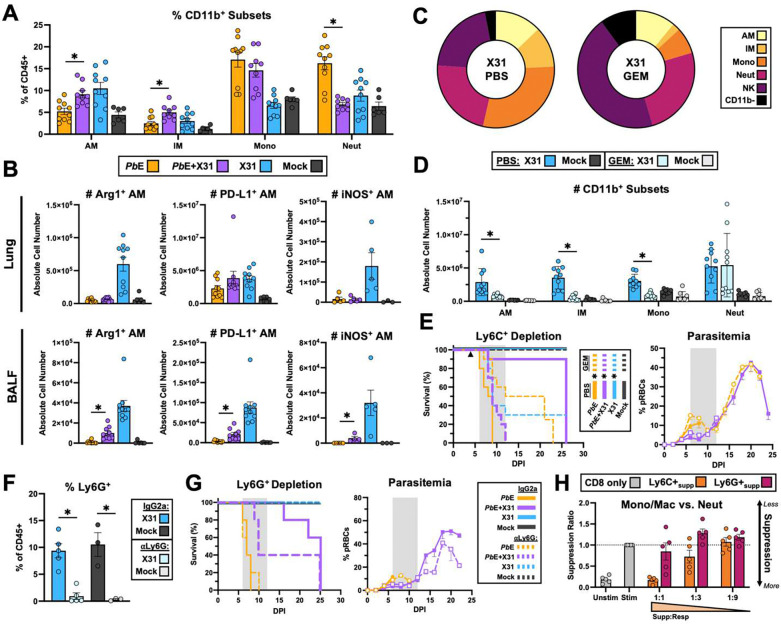
Ly6C^+^ cells are required for the protective effect of X31 on *Pb*E-mediated MA-ALI. Co-infected mice had a higher frequency of AMs (p=0.004), IMs (p=0.002), and a lower frequency of neutrophils (p<0.0001) than *Pb*E singly-infected mice at 6 DPI (Mann-Whitney U) (n=6–10 mice per group) **(A)**. The number of Arg1^+^, PD-L1^+^, and iNOS^+^ AMs are all significantly increased in the BALF of co-infected mice relative to *Pb*E singly-infected mice (Arg1 p<0.0001, PD-L1 p=0.095, iNOS p=0.016; Mann-Whitney U), but not in the lung tissue (Mann-Whitney U all P>0.05) (n=6–10 mice per group) **(B)**. Relative frequencies **(C)** and absolute cell number **(D)** of CD45^+^ cells in X31-infected mice at 6 DPI in GEM- treated mice compared to PBS-treated mice, show that GEM administration successfully removes the Ly6C^+^ subsets from the lung (all p<0.0001; Mann-Whitney U) (n= 6–10 mice per group). Upon removal of Ly6C+ subsets, co-infected mice died 8–12 DPI, significantly faster than co-infected mice treated with PBS (p<0.001) with increased survival in *Pb*E singly-infected mice (p=0.0014) and increase death in X31 singly-infected mice (p=0.0012) (Kaplan Meier Analysis with Mantel-Cox Test and Holm-Bonferroni correction for multiple comparisons) (n=6–10 mice per group) **(E)**. The grey box depicts the window when mice die from MA-ALI. GEM treatment did not impact parasitemia early parasitemia in *Pb*E-singly infected or co-infected mice (Kruskal-Wallis of AUC 0–6 DPI both p>0.05). Depletion of neutrophils (**F**: X31 p=0.0278; Mock p=0.0410; Kruskal-Wallis pairwise mean rank comparisons GEM vs. PBS; n=3–5 mice per group) did not alter the survival from *Pb*E or X31 infection s (p-0.804 or P>0.99 respectively) but did reduce the survival of co-infected mice from MA-ALI in days 6–12 (P=0.05), albeit all mice ended up succumbing from infection at day 25 DPI (p=0.346) (Kaplan Meier Analysis with Mantel-Cox Test and Holm-Bonferroni correction for multiple comparisons: αLy6G vs. IgG2a) (**G**). Neutrophil depletion did not impact parasitemia in *Pb*E single infected mice (p>0.999) or co-infected mice (p=0.269) (Kruskal-Wallis of AUC 0–6 DPI). Culture of Ly6C+ or Ly6G+ pulmonary derived cells from X31-infected mice at 6 DPI with stimulated CTV-stained CD8 T cells (n=5 biological replicates per group) demonstrates that Ly6G+ neutrophils have minimal suppressive activity for CD8 T cell proliferation *in vitro* (p=0.0079; Mann-Whitney U: Ly6C^+^_supp_ 1:1 vs. Ly6G^+^_supp_ 1:1) (H). Data are represented as mean ± SEM.

There was significant AM expansion in *Pb*E+X31 co-infected mice compared to *Pb*E singly-infected mice (Figure 4A). While AMs expressing Arg1, PD-L1, and iNOS did not greatly expand in the lung tissue of co-infected mice, there was a significant increase in the number of AMs expressing Arg1, iNOS and PDL-1 in the BALF compared to *Pb*E singly-infected mice (Figure 4B, Supplemental Figure 5A). To determine if X31-induced monocytes/macrophages were necessary for protection against *Pb*E-induced MA-ALI, we depleted these cells using the nucleoside analog gemcitabine (GEM). This drug is a proposed cancer therapeutic that selectively depletes Ly6C^+^ cells.^44^ After treating mice with 1.2 mg GEM i.p. at 4 DPI, depletion of Ly6C^+^ cells in X31m singly-infected mice led to a decreased in AMs, IMs, and monocytes relative to untreated X31singly-infected mice (Figure 4C). While neutrophils were a larger proportion of CD45^+^ cells after GEM treatment, they were not different in number (Figure 4D), and the number of T cells (CD4^+^ and CD8^+^), CD11b^−^, NK, and B cells were not impacted (Supplemental Figure 5B). Upon depletion of Ly6C^+^ cells X31 infection. was no longer able to protect against *Pb*E-induced MA-ALI (Figure 4E). Interestingly depletion of Ly6C^+^ cells led to a partial increase in survival in mice infected with *Pb*E alone, possibly due to the depletion of pathogenic inflammatory Ly6C^+^ cells in malaria^45^ (Figure 4B). We also determined that ablation of Ly6C^+^ cells transformed a previously non-lethal X31 infection into an infection, with a survival rate of only 30% (Figure 4E) demonstrating that Ly6C^+^ cells expressing suppressive molecules are elicited by X31 to protect against influenza-induced pulmonary immunopathogenesis.

Neutrophils were also significantly increased in *Pb*E singly-infected mice but not in co-infected mice (Figure 4A). Although X31-induced neutrophils express very few suppressive molecules (Figure 2D), a portion (~12%) express PD-L1. Neutrophils have also been reported to have suppressive abilities in tumor models^46, 47^. We used an αLy6G antibody with a Mar18.5 enhancer to ablate neutrophils in C57BL/6 mice (Figure 4F) and, although unexpected, neutrophil depletion resulted in a partial reversal of the co-infection protective phenotype (60% death at 6–12 DPI), indicating that neutrophils also play a role in protection. However *in vitro*, X31-induced neutrophils did not suppress CD8 T cell proliferation as effectively as X31-induced Ly6C^+^ cells (Figure 4H) and, if anything, enhanced the proliferation at lower ratios. Together, these data show that both neutrophils and Ly6C^+^ monocyte/macrophage subsets play a role in X31-induced protection from *Pb*E-induced MA-ALI but the mechanism by which neutrophils exert protection may differ from that of Ly6C^+^ cells.

### Pulmonary CD8 T cells exhibit phenotypic deficiencies in co-infection.

**Figure 5.**
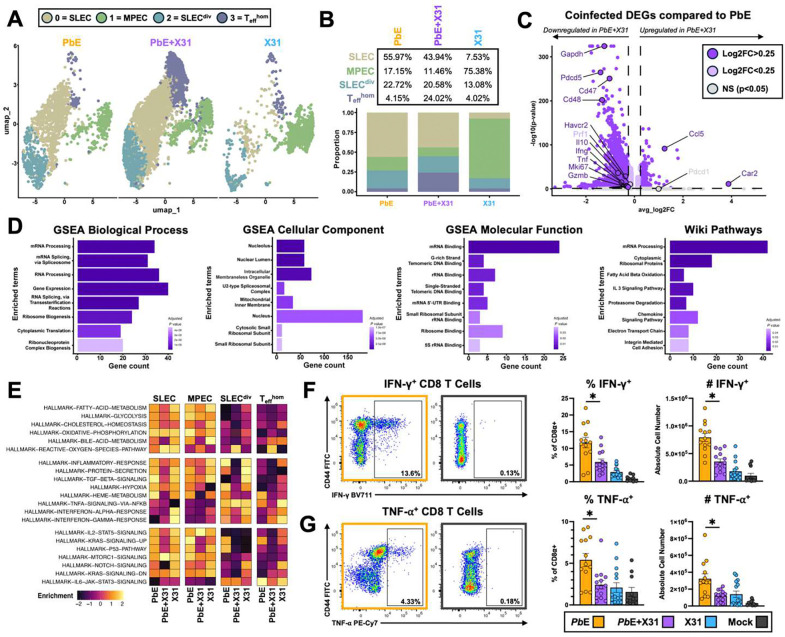
X31 co-infection reduces the pathogenicity of CD8 T cells recruited by *Pb*E infection. Single-cell RNA sequencing analysis of pulmonary CD8 T cells from *Pb*E, *Pb*E+X31, and X31 mice at 6 DPI (n = 3 mice pooled per group) results in four clusters: short-lived effector cells (SLEC), memory progenitor effector cells (MPEC), dividing SLECs (SLEC^div^), and homing T effector cells (T_eff_^hom^) **(A)**. While MPEC, and SLEC^div^ are similar between *Pb*E and *Pb*E+X31 infected mice, T_eff_^hom^ (purple) is expanded in the co-infected group at the expense of SLECs (beige) **(B)**. Volcano plot showing differentially expressed genes (DEGs) in activated CD44+ CD8 T cells obtained from the lung tissue of *Pb*E+X31 co-infected vs. *Pb*E singly-infected mice at 6 DPI (log_2_Fc cutoff=0.25, −log_10_p-value=0.05) **(C)**. A large proportion of genes are downregulated in co-infected mice compared to *Pb*E singly-infected mice. Two genes extend beyond the right of the x-axis: lgkv8–27 (log_2_Fc=7.385, p_adj_<0.001) and lghv9–3 (log_2_Fc=7.007, p_adj_=1.0). Downregulated gene set enrichment analysis (GSEA) for biological processes, cellular components, molecular function, and Wiki pathways in CD8 T cells from X31+*Pb*E co-infected mice relative to *Pb*E singly-infected mice. **(D)**. Gene set variation analysis (GSVA) of metabolic and effector gene pathways for each CD8 T cell subset. Gene are downregulated in several pathways in CD8 T cells from *Pb*E+X31 coinfected mice relative to *Pb*E singly-infected mice **(E)**. There are fewer IFN-γ^+^ (**F**: % p=0.008; # p<0.001) and TNF^+^ (**G**: % p=0.004; # p=0.003) CD8 T cells by both frequency and absolute cell number in co-infected mice compared to *Pb*E singly-infected mice (Mann-Whitney U). Data are represented as mean ± SEM.

Since *Pb*E-induced MA-ALI is a CD8 T cell-mediated disease and recruitment of *Pb*E-reactive CD8 T cells was not significantly reduced during co-infection (Fig 1D–E), we hypothesized that X31 incuded myeloid cells may mediate protection by reducing the pathogenicity of *Pb*E-induced CD8 T cells. We performed single-cell RNA sequencing analysis (scRNAseq) on pulmonary CD8 T cells from X31+*Pb*E co-infected and *Pb*E singly-infected mice at 6 DPI (Supplemental Figure 6A). CD8 T cells clustered into four populations that were identified as short-lived effector cells (SLEC; Cluster 0), memory progenitor effector cells (MPEC; Cluster 1), dividing SLECS (SLEC^div^; Cluster 2), and homing T effector cells (T_eff_^hom^) (Figure 5A, Supplemental Figure 6B–C). While the relative proportions of SLEC, MPEC, and SLEC^div^ in the lung tissue of *Pb*E-infected mice were not significantly impaired by X31 infection, the T_eff_^hom^ population was expanded in co-infected mice relative to *Pb*E singly-infected mice at the expense of SLECs (Figure 5B). Comparison of differentially-expressed genes (DEGs) revealed that many genes are significantly downregulated in CD8 T cells from protected co-infected mice compared to those with lethal *Pb*E infection including genes encoding IFN-γ (*Ifng*), TNF-α (*Tnf*), Granzyme B (*GzmB*), proliferation marker Ki-67 (*Mki67*), Tim-3 (*Havcr2*), and glyceraldehyde-3-phosphate dehydrogenase (*Gapdh*) (Figure 5C). Gene set enrichment analysis (GSEA) of downregulated genes in CD8 T cells from co-infected mice highlight pathways in transcription, translation, mitochondrial function, and metabolism (Figure 5D), signifying deficits in CD8 T cell function. Gene set variation analysis (GSVA) of individual clusters shows that CD8 T cells from co-infected mice across all populations are downregulated in functional pathways relating to metabolism, inflammatory responses, and signaling relative *Pb*E singly-infected mice (Figure 5E). The SLEC^div^ cluster is particularly impaired in protected co-infected mice relative to susceptible *Pb*E singly-infected mice. We confirmed that there are significantly fewer IFN-γ ^+^ and TNF-α ^+^ CD8 T cells by frequency and number in the lung tissue of co-infected mice compared to *Pb*E singly-infected mice (Figure 5F–G), validating the deficiencies highlighted by the scRNAseq analysis. Together, these data demonstrate that pulmonary CD8 T cells experience a significant reduction in their effector functions during co-infection leading to X31-mediated protection from MA-ALI.

## Discussion

This study reports a novel co-infection dynamic in which a virus-induced, tissue-restricted immunosuppressive myeloid cell population can suppress pathogenic CD8 T cells to protect against malaria-induced lung pathology. This protection does not arise from impaired CD8 T cell recruitment to the site of inflammation (Figure 1). Rather, the phenotype reflects tissue-specific dampening of CD8 T cell effector capacity, characterized by transcriptional repression and significant deficits in cytokine production. Clinically this finding is highly significant because it demonstrates that pathology can be interrupted even after CD8 T cell priming and tissue infiltration, as would be the case in MA-ARDS / MA-ALI patients that seek medical care after respiratory symptoms develop.

Whilst it has been reported that *Plasmodium* infected individuals who were seropositive for SARS-CoV-2 did not appear to have increased progression to developing malaria relative to seronegative individuals ^23^ there are very few studies that examine the effects of concomitant respiratory viral infections on MA-ALI. Our findings suggest that interactions between unrelated pathogens can reshape tissue-specific immunity in ways that fundamentally alter disease trajectory, highlighting co-infection as both a source of risk and an underappreciated opportunity for immune modulation.

Mice infected with *Pb*E are unable to durably control parasite burden in the absence of chemotherapeutic intervention and therefore succumb to hyperparasitemia if organ-specific pathologies are avoided. As such, the extended survival observed during co-infection reflects mitigation of immunopathology, rather than sterilizing or long-term parasite control. The high prevalence of asymptomatic infections that can occur at varying levels of circulating parasitemia in younger individuals^48^ demonstrates that innate immune mechanisms can control of *Plasmodium*-associated immunopathogenesis and disease in the face of parasite-induced inflammation.

The temporal requirement for protection supports a model in which early innate immune cell programming, rather than established adaptive responses, imposes a transient environment that drives the inhibition of pathogenic CD8 T cells. We identified a population of Ly6C^+^ monocyte-derived alveolar macrophages that are required for protection and suppress CD8 T cell activation in a contact-dependent manner. These cells express inhibitory markers (Arg1, PD-L1, iNOS) during their response in the lung following X31 infection and, yet none of these molecules were individually required for suppression *in vitro*, suggesting a redundant or multidimensional suppressive mechanism. Suppressive macrophages in tumor environments are heterogeneous^49^ and inhibit immune responses via mechanisms that involve a variety of molecules^50^ including the contact dependent molecules Fas ligand (FasL)^51^ and CTLA4^52^. The broad transcriptional suppression observed in CD8 T cells implicates a model in which X31-induced myeloid cells use multiple molecules to impose a broad immunosuppressive environment on the lung that limits immunopathology.

Our data demonstrate that this protective myeloid response is strain dependent. We have used this observation to pinpoint the mechanism by which X31 protects against MA-ALI in mice. We determined that while type I IFN signaling contributes to the expansion of macrophages during infection, it is not sufficient to explain their suppressive phenotype, particularly given that PR8 canonically induces higher levels of type 1 interferons. These findings suggest that differential host-pathogen interactions that may be potentially mediated by variation in viral surface proteins or cell tropism between strains shapes early inflammation signaling and the qualitative features of the innate response. Future studies must explore these findings in the context of human disease, where influenza is highly heterogeneous and may vary substantially across settings^53^. Even if clinical isolates of influenza fail to produce the same suppressive myeloid response, murine X31 infection provides a powerful system to study the induction of these cells.

Myeloid cell induced suppression of CD8 T cells are seen as a major barrier to therapeutic efficacy against tumors^54^ where CD8 T cell lytic capability is a key component of tumor control. The involvement of myeloid cells with suppressive activity in infectious disease contexts is much more limited^55^. We are not the first to describe the expansion of myeloid cells with suppressive activity in viral infection. Expansion in response to hepatitis virus, HIV, SARS-CoV-2^55^ and Japanese Encephailitis Virus^56^ infections are almost uniformly detrimental with respect to suppression of CD8 T cell-mediated viral control. X31-mediated protection from *Pb*E pulmonary leak shows how suppressive programs can be protective by limiting immune-mediated tissue damage to an unrelated infection.

Our data demonstrates that immunosuppression is not inherently pathological but can be protective when appropriately localized and temporally constrained. The significant body of research on the induction of suppressive myeloid cells in tumors to prevent their expansion could be conversely exploited to induce these regulatory cells during severe malaria. Therapeutic strategies that transiently induce or mimic this suppressive myeloid state could reduce mortality from MA-ALI and related complications.

## Material and methods

### Mice and infections

All animals were housed in specific-pathogen-free conditions, and all work was performed in accordance with Institutional Animal Care and Use Committee (IACUC) protocol #00002078. Experiments were carried out in female C57BL6/J mice (Jackson Laboratories #000664) aged 6–8 weeks. *Plasmodium berghei* NK65-Edinburgh (*Pb*E) was a kind gift from Dr. Philippe Van den Steen (KU Leuven). Infections were established with intraperitoneal (i.p.) injection of 5×10^5^ iRBCs from donor blood diluted in Krebs-Ringer solution (ThermoFisher #J67591.AP) in a volume of 200 μl. Influenza/A/X31 and Influenza/A/PR8 lines was a kind gift from Dr. Jacob Kohlmeier (Emory University). Frozen stocks were diluted in 1X phosphate buffered saline (PBS; Gibco #10010–023) and experimental mice were intranasally (i.n.) inoculated under isoflurane anesthesia with 3×10^4^ plaque forming units (PFU) of Influenza/A/X31. Influenza/A/PR8 infections were at sublethal doses of 35 PFU. Live-attenuated PR8 (PR8-LAIV; a kind gift from Drs. Jacob Kohlmeier and Anice Lowen, Emory University) was administered in the same manner as live-virus infections but at a dose of 5×10^4^ PFU. In some experiments, the influenza virus was heat-killed by incubating on a heat block at 56°C for 30 minutes before inoculation.

Parasitemia was evaluated by staining 1:25 dilution of peripheral blood with CD45-APC (1:200, Biolegend #109813), CD71-PE (1:200, Biolegend #113807), Hoechst 34580 (1:200, BD Biosciences #565877) for 30 minutes at 4°C prior to analysis by flow cytometry (X20 analyzer, BD Biosciences). iRBCs were identified as CD45^−^ Hoechst^+^ and parasitemia was confirmed by microscopy of blood smears. Mice were monitored daily for survival. Every other day, mice were sampled for health measures including weight loss, RBC loss, and parasitemia.

### Isolation of single cell suspensions

Tissues were collected into 1 mL incomplete RPMI (iRPMI; RPMI 1640 (Corning10–040-CV) with 200 mM L-glutamine, 1X penicillin/streptomycin + 4 mM L-glutamine). Spleens were homogenized by pushing the tissue through a 100 μm cell strainer with a syringe plunger. Lung tissue was placed into a gentleMACS C Tube containing 5 mL digestion media (5 mL iRPMI, 2 mg/mL Collagenase D, 20 g/mL DNase I). Lungs were minced in a Miltenyi gentleMACS machine using program m_lung_01, incubated for 30 minutes on a shaker at 37°C, and homogenized using gentleMACS program m_lung_02. Lung homogenates were filtered through a 100 μm cell strainer and centrifuged at 300 × g for 5 minutes. Red blood cells were removed by incubating with 1X RBC Lysis Buffer (eBioscience 00–4333-57) for 1 minute at room temperature before 9 mL complete RPMI (cRPMI; RPMI 1640 with the addition of 200 mM L-glutamine 10% FBS, 1X penicillin/streptomycin + 4 mM L-glutamine, 0.01 M HEPES, 0.05 mM 2-β-mercaptoethanol). Following centrifugation and removal of the supernatant, homogenates were resuspended in cRPMI and filtered again. Cell counts were obtained as described above.

### Flow cytometry

2×10^6^ cells per sample were transferred to a 96-well plate and washed with MACS buffer (D-PBS, 0.5% BSA, 2 mM EDTA). Samples were incubated at room temperature with 50 μL of Fc block (αCD16/CD32, 1:100, Biolegend #101319) for 20 minutes before staining dead cells with the Zombie UV Fixable Viability Stain (1:1000, Biolegend #423105). Samples were stained for surface markers for 30 minutes at 4°C, followed by fixation/permeabilization and intracellular staining for 1 hour at 4°C (Supplementary Table 1). For intracellular cytokine staining, lung homogenates were incubated at 37°C with Brefeldin A for 4 hours prior to surface staining. During analysis, debris, doublets, and dead cells were excluded before gating to identify monocytes (Mono; CD45^+^ TCRβ^−^ CD19^−^ NK1.1^−^ CD11b^+^ Ly6C^+^ CD11c^−^ MHCII^−^), interstitial macrophages (IM; CD45^+^ TCRβ^−^ CD19^−^ NK1.1^−^ CD11b^+^ Ly6C^+^ CD11c^−^ MHCII^+^ F4/80^+^ CD64^+^), monocyte-derived alveolar macrophages (AM; CD45^+^ TCRβ^−^ CD19^−^ NK1.1^−^ CD11b^+^ Ly6C^+^ CD11c^+^ F4/80^+^ CD64^+^), and neutrophils (Neut; CD45^+^ TCRβ^−^ CD19^−^ NK1.1^−^ CD11b^+^ Ly6G^+^). A complete gating strategy can be viewed in Supplemental Figure 2.

### Myeloid Cell Depletions

Mice were intraperitoneally (i.p.) injected with 12 mg/kg Gemcitabine (GEM, Sigma Alrich #G6423) in a volume of 200 μL at 4 DPI and were monitored for survival or euthanized at 6 DPI to confirm depletion efficacy by flow cytometry. Neutrophils were depleted by IP injecting mice with 25 μg of αLy6G (clone 1A8, BioXCell # BP0075–1) or IgG2a (clone 2A3, BioXCell # BP0089) every other day beginning at 0 DPI^57^. On the alternating days (1, 3, 5, etc. DPI), all mice were injected with a 50 μg Kappa Immunoglobulin Light Chain enhancer antibody (clone MAR18.5, BioXCell # BE0122)^58^. Depletion efficacy was determined by flow cytometry of isolated pulmonary cells at 6 DPI as described below.

### FITC-Dextran Permeability Assay

70 kDA fluorescein (FITC)-conjugated dextran (ThermoFisher #D1823) was resuspended in 1X PBS and filter sterilized. At 6 DPI, mice were intravenously (i.v.) injected with 2 mg FITC-Dextran solution in a volume of 100 μL. Bronchoalveolar lavage fluid (BALF) was collected from mice by inserting an 18 G catheter into the trachea and flushing the lungs twice with 750 μL 1X PBS each time (1.5 mL total). The BALF was centrifuged at 2000 × g for 10 minutes and the supernatant collected. 100 μL of BALF was transferred to a 96-well flat-bottomed, black-walled plate. Fluorescence (Ex/Em 482/525) was analyzed on a plate reader (Biotek Synergy) and quantified by comparing to a standard curve.

### MicroBCA Assay

Following euthanasia at 6 DPI, BALF was collected in the same manner as described above. 150 μL of each BALF sample was aliquoted in duplicate and a microBCA protein assay was performed using the ThermoFisher Scientific kit #23235. The color change indicating protein concentration was quantified on undiluted and diluted (1:3) samples using a plate reader (Biotek Synergy) at an absorbance of 562 nm. Protein levels were quantified by comparing to a bovine serum albumin (BSA) standard curve provided in the kit.

### Suppression assays

Spleens from naïve mice were harvested and processed into single-cell suspensions as described above. CD8α ^+^ T cells were isolated via magnetic-activated cell separation (MACS, Miltenyi #130–104-075). Cells were stained with CellTrace Violet (Invitrogen #C34557) for 20 minutes at 37°C and resuspended in cRPMI supplemented with 5 ng/mL recombinant mouse interleukin 2 (IL-2; Biolegend #575404). 5×10^5^ cells/well were plated in a 96-well round-bottom plate or a 96-well 0.4 M-pore transwell plate. Mouse T-cell activator CD3/CD28 Dynabeads (Gibco #11–452-D) were washed with PBS, resuspended in IL-2-supplemented cRPMI, and plated at a concentration of 5×10^5^ beads/well.

Lungs of Influenza/A/X31-infected mice were harvested at 6 DPI and processed into homogenates as described above. Ly6C^+^ cells and Ly6G+ cells were isolated via MACS using a mouse Myeloid-Derived Suppressor Cell Isolation Kit (Miltenyi #130–094-538), which purifies both Gr1^dim^Ly6G^−^ (monocyte/macrophage) and Gr1^hi^Ly6G^+^ (neutrophil) populations. Isolated myeloid populations were resuspended in IL-2-supplemented cRPMI and plated in duplicate with the responder CD8 T cells at ratios of 1:1, 1:3, and 1:9.

An aliquot of each cell population was taken during plating and stained for flow cytometry as described above to confirm purity of the isolation and the CTV staining of the responder cells. Plates containing myeloid-responder cocultures were incubated at 37°C with 5% CO_2_ for 48 hours. The cells were then centrifuged at 300 × g for 5 minutes and transferred to 96-well V-bottom plates before staining for flow cytometry using the method described above. To assess the level of baseline activation and proliferation, wells containing unstimulated CD8 T cells alone were used as an unproliferated control, while wells containing stimulated CD8 T cells alone were used as a proliferation control. To account for possible stimulation differences between CD8 T cells between experiments the results are reported as a suppression score, which is equal to (% proliferated in the experimental group) / (% proliferated in the CD8 stimulated group).

To test whether the suppression of CD8 T cells was contact-dependent, we performed a suppression assay using conditioned media. Myeloid cells and responder cells were isolated, prepared, and co-cultured in the same manner as described above. After 12 and 24 hours of culturing, the cells were centrifuged at 300 × *g* for 5 minutes. The supernatant, which would contain possibly suppressive soluble factors secreted by the myeloid cells, was collected and placed in wells containing freshly-isolated CD8 T cells and stimulation beads. 48 hours after the media was added to the the fresh CD8 cells, flow cytometry was performed as described to measure activation and proliferation.

We added several inhibitors to wells containing 1:1 CD8 : Ly6C^+^ cells to test the necessity of different suppressive molecules on CD8 T cell inhibition. To inhibit Arg1, 0.10–100 M of Nω-Hydroxy-nor-L-arginine (norNOHA, MCE #HY-112885B/CS-0121051) was added, with DMSO as a control. To test iNOS, 0.01–1000 M of Nω-Nitro-l-arginine methyl ester hydrochloride (L-NAME, Millipore Sigma #483125) was added, with dH_2_O (0.4% final concentration) as a control. To test PD-L1/PDL2, 0.16–10 μg of αPD-L1 (clone 10F.9G2TM, BioXCell #BE0101) and αPD-L2 (clone TY25, BioXCell #BE0112, with IgG2b (clone LTF-2-CP116, BioXCell #BE0090) and IgG2a (clone 2A3, BioXCell #BE0089) as isotype controls. The test the requirement of PDL1 expression on Ly6C+ cells for suppression, a suppression assay was undertaken as above but comparing proliferation of WT (C57BL/6J) and PD1^−/−^ (Jackson Laboratories # 028276) CD8 T cells.

### 2D Micropipette Adhesion Frequency Assay (2D-MP)

Lung homogenates incubated with Fc block, live-dead stain, and surface markers (Supplementary Table 3) in the manner described above. Activated CD8 T cells (ZombieNIR^−^ TCRβ^+^ CD4^−^ CD8^+^ CD44^+^) were sorted on a Sony MA900 Cell Sorter with a 100 μm chip.

The 2D-MP assay was performed as previously described^30–32^. In short, human RBCs were coated with monomers for H^2^-D^b^:GAP50_40–48_ (*Plasmodium*)^25^, H^2^-D^b^:NP_366–374_ (Influenza)^59^ or H^2^-D^b^:GP_33–41_ (lymphocytic choriomeningitis virus, LCMV)^60^ at several known densities between 1–100 μM. All monomers were provided by the National Institutes of Health Tetramer Core Facility at Emory University.

Coated RBCs and isolated CD8 T cells were loaded into separate zones of the microscope chamber. A CD8 T cell was selected at random and brought into contact with a peptide-MHC-coated RBC for 2 seconds, beginning with the highest coating of the antigen of interest. Binding events were identified by distention of the RBC membrane upon retraction of the CD8 T cell. If the adhesion frequency (*P*_a_) was < 0.1, the T cell was considered non-reactive to that antigen. Cells with a *P*_a_ > 0.8 were tested against a lower density coating RBC to ensure measurement accuracy. The percent of antigen-reactive cells was calculated by (# T Cells with *P*^a^ > 0.1) / (Total # T Cells Tested). Cells with a *P*_a_ between 0.1–0.8 were subjected to 50 contacts with the RBC at the appropriate peptide-MHC density to determine the TCR affinity for the antigen. Relative 2D affinity (K_a_) was calculated with the equation A_c_K_a_(μm^4^) = −ln(*P*_a_)/(ρ_TCR_ × ρ_pMHC_), wherein A_c_ is the contact area, and ρ_TCR_ and ρ_MHC_ denote the densities of the TCR and MHC on the CD8 T cells and RBCs respectively. The geometric mean fluorescence intensities of the TCR and peptide-loaded MHC (PE-H^2^-D^b^, Clone 28–14-8, 1:50 dilution) and Quantibrite PE Beads (BD Biosciences #340495) are run through a linear regression model to quantify receptor densities.

CD8 T cells were tested against both GAP50^40–48^ and NP^366–374^. If a cell was cross-reactive against both epitopes, it was also tested against GP^33–41^. In the case of single infections, CD8 T cells were tested against the primary epitope for the respective infection and reactive cells were tested against the other epitopes. Cross-reactive cells were excluded from the primary analysis. The binding frequency represents the proportion of cells tested that specifically-bound the epitope of interest. Estimated antigen-reactive cell numbers were calculated by multiplying the absolute cell number of CD8^+^ CD44^+^ cells by the binding frequency.

### Single-cell RNA Sequencing

Bulk T cells were isolated from lung tissue via MACS using the Miltenyi Pan T Cell Isolation Kit II, mouse (#130–095-130). Cells were then diluted to 1000 cells/μL before being run through the 10x chromium pipeline (10x Genomics). 10,000 cells were targeted and between 4,500 and 16,000 cells were recovered after library preparation was completed. Libraries were constructed at the University of Utah DNA Sequencing Core as per the 5’ gene expression recommended protocol (10x Genomics). Libraries were sequenced on an Element Aviti targeting 20,000 reads per cell. Bioinformatic analysis was completed using 10X Genomics CellRanger 6.0 and Seurat v5.4.0 in R v4.5.2.^61, 62^ Briefly, doublets and dead cells were removed, and clustering was completed using the standard Seurat v5.4.0 pipeline. Each individual sample was assessed for mitochondrial RNA and any cell with more than 10% was removed. Samples were then merged, normalized, scaled, and integrated to allow direct comparisons between the samples. Once clusters were determined by the Louvain clustering algorithm in Seurat v5.4.0, CD8 T cells were identified, isolated, and re-clustered into their respective populations. Differential gene expression analysis was completed between samples using the CD8α^+^ clusters identified using Seurat v5.4.0 standard protocols. Further analysis of metabolic and functional pathways was done using scRepertoire 2.6.2 and escape 2.6.2^63^. Plots were generated using the ggPlot2 4.0.1 interface with Seurat v5.4.0 and EnhancedVolcano 1.28.2. Raw sequencing files as well as post-processed matrix files are publicly available under GEO Accession Number: GSE323359.

### Statistical analysis

A Kaplan Meier Analysis with Mantel-Cox Test and Holm-Bonferroni correction for multiple comparisons was used to test for differences in survival. For cumulative parasitemia, the area under the curve (AUC) was generated for each mouse before group differences were analyzed by Kruskal-Wallis. For flow cytometry comparisons, all data was analyzed via Mann-Whitney U test (for 2 groups) or Kruskal-Wallis (for 3+ groups). A p-value of < 0.05 was considered statistically significant. All statistical analysis was performed using Graphpad Prism software.

## Supplementary Material

Supplement 1

Supplementary Files

This is a list of supplementary files associated with this preprint. Click to download.

• SupplementalFigures.xlsx
