# Supplementary material for: Protective coinfection: influenza reprograms myeloid cells to limit CD8 T cell–mediated malaria pathology
Source: Res Sq. 2026 May 8:rs.3.rs-9404453. Preprint. [Version 1] doi: 10.21203/rs.3.rs-9404453/v1 (PMC13174795; doi:10.21203/rs.3.rs-9404453/v1)
Supplement: Supplement 1 [file NIHPPRS9404453V1-supplement-1.pdf]

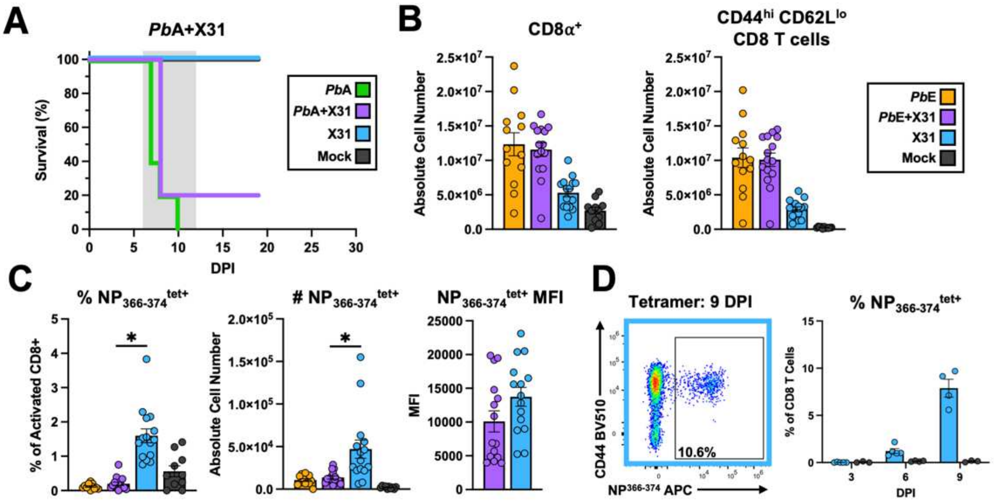

Supplemental Figure 1. Extended data on the X31 mediated protection against *Pb*E-induced MA-ALI. Survival was not significantly different between coinfected and *Pb*A singly-infected mice (p=0.147; Kaplan-Meier Analysis with Mantel-Cox Test) (n=5 mice per group) **(A)**. There was no difference in the number of total CD8 T cells (p=0.7767; Mann-Whitney U) or activated ($CD44^{hi}$ $CD62L^{lo}$) CD8 T cells (p=0.9728, Mann-Whitney U) between coinfected and *Pb*E-singly-infected mice at 6 DPI (n=11-15 mice per group) **(B)**. X31-singly-infected mice had more high-affinity $NP_{366\text{-}374}$-reactive cells than co-infected mice (% p<0.001, # p=0.0008, MFI p=0.0675; Mann-Whitney U) **(C)**. There was not a significant $NP_{366\text{-}374}^{tet+}$ population in the lungs of mice singly-infected with X31 at 6 DPI but the population was visibly expanded by 9 DPI **(D)**. Data are represented as mean ± SEM.

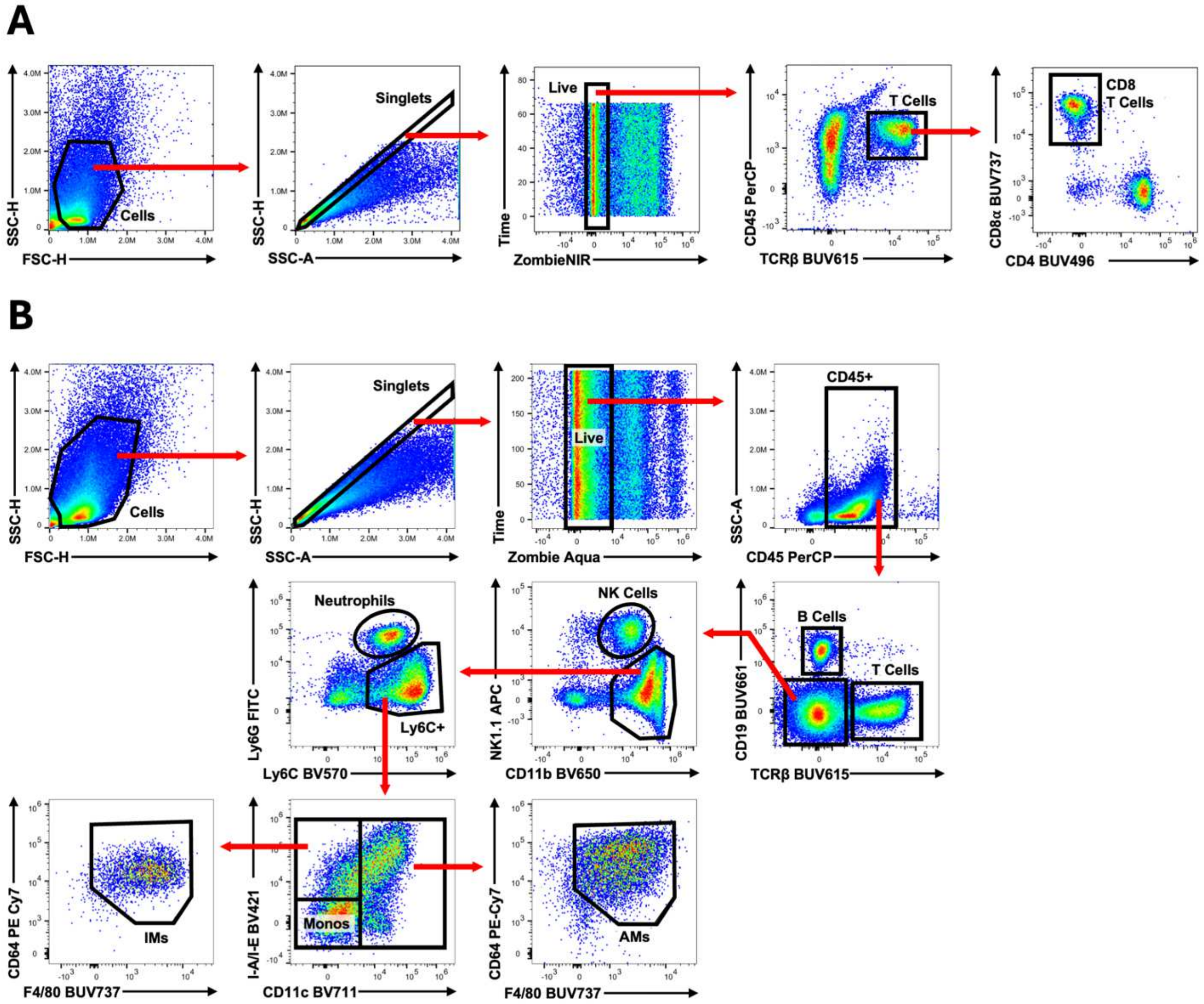

Supplemental Figure 2. Flow cytometry gating strategies. CD8 T cells were identified as $CD45^+$ $TCR\beta^+$ $CD8\alpha^+$ $CD4^-$. Tetramer and cytokine analysis was immediately downstream of the $CD8^+$ gate **(A)**. After gating out debris, doublets, and dead cells, myeloid cells were identified by gating $CD45^+$ $TCR\beta^-$ $CD19^-$ **(B)**. Subsequently, NK cells were separated from the other $CD11b^+$ populations, which were further gated into $Ly6G^+$ $Ly6C^{mid}$ neutrophils and $Ly6G^-$ $Ly6C^+$ monocytic cells. From the $Ly6C^+$ population, monocytes were identified as $CD11c^-$ MHC-II^-, interstitial macrophages were identified as $CD11c^-$ MHC-II^+ F4/80^+ $CD64^+$, and monocyte-derived alveolar macrophages were identified as $CD11c^+$ F4/80^+ $CD64^+$. The suppressive markers Arg1, PD-L1, and iNOS were gated following terminal gating of each subset.

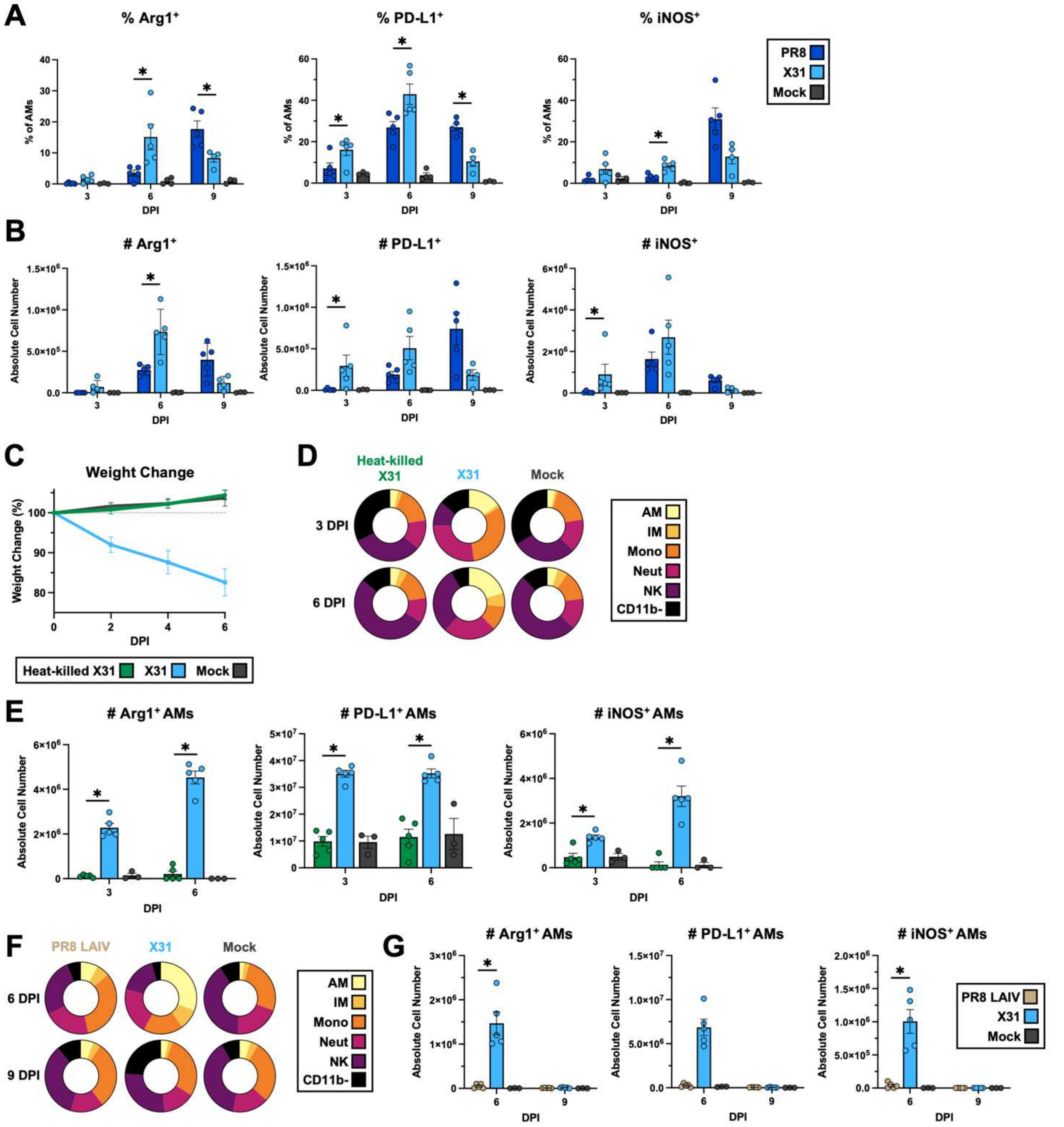

Supplemental Figure 3. Live X31 viral infection induces more myeloid cells expressing suppressive molecules than PR8 infection. The frequency **(A)** and absolute cell numbers **(B)** of Arg1$^+$ AMs (D6 % p= 0.024; absolute cell number p=0.024), PDL1$^+$ AMs (D3 % p =0.047; absolute cell number p=0.047), and iNOS$^+$ AMs (D3 % p=0.151; absolute cell number p=0.024) is more substantial in X31 infection relative to than in PR8 infection (Mann-Whitney U) (n= 3-5 mice per group). Mice that received heat-killed X31 (green) fail to lose weight compared to the live X31 group (blue) **(C)** and there was no expansion of CD11b$^+$ subsets (AM, IM, Mono, Neut) **(D).** Expansion of Arg1$^+$ (both time points p=0.008), iNOS$^+$ (D3 p=0.016, D6 p=0.008) or PD-L1$^+$ (both time points p=0.008) in AMs was only observed during infection with live X31 (Mann-Whitney U: Heat-killed vs Live) **(E)**. High dose (5x10^4 PFU) PR8 LAIV fails to expand AMs expressing suppressive molecules to the same extent as X31 (n = 3-5 mice per group) **(F)**. PR8 LAIV did not induce the expansion of Arg1$^+$ (D6 p= 0.008,), PD-L1$^+$ (D6 p=0.008), or iNOS$^+$ (D6 p=0.008) expressing AMs (Mann-

Whitney U: PR8 LAIV vs. X31) **(G)**. Data are represented as mean ± SEM. AM: alveolar macrophage; IM: interstitial macrophage; Mono: monocyte; Neut: neutrophil; NK: natural killer cell

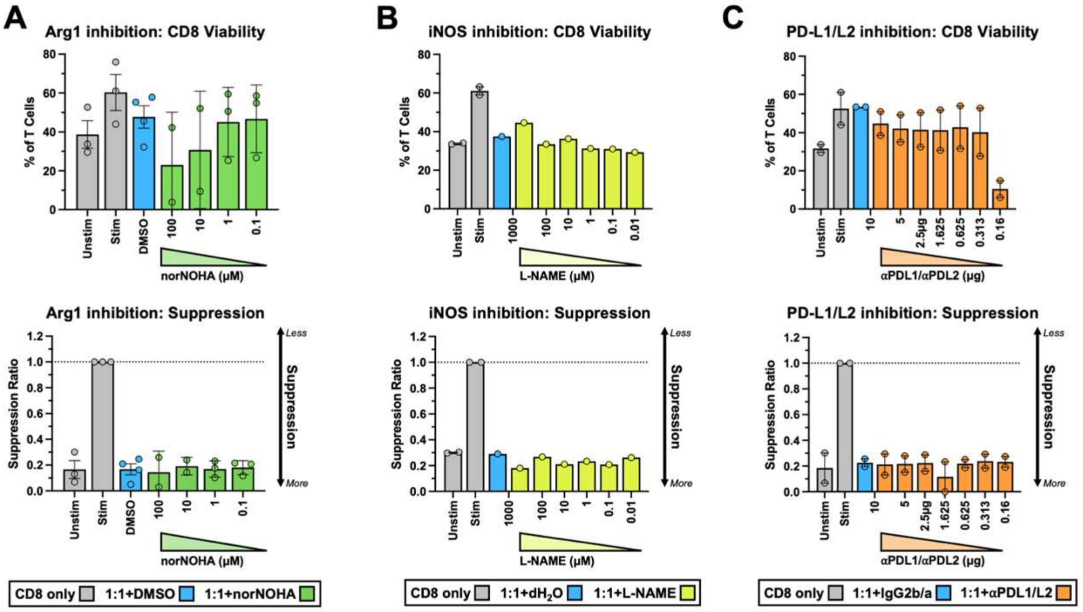

Supplemental Figure 4. The suppressive ability of X31-induced pulmonary Ly6C⁺ cells does not depend on Arg1, iNOS or PD-L1/L2 *in vitro*. Suppression assays were set up with stimulated CD8 T cells and pulmonary Ly6C⁺ cells from X31-infected mice at 6

DPI. **(A)** CD8 T cell viability (left) and suppression ratio (right) for 1:1 CD8:Ly6C$^+$ cells cultured with DMSO (blue) or 100-0.1 µM norNOHA (green) to inhibit Arg1 activity. **(B)** CD8$^+$ T cell viability (left) and suppression ratio (right) for 1:1 CD8: Ly6C+ cells cultured with dH_2O (blue) or 1000-0.01 µM L-NAME (orange) to inhibit iNOS activity. **(C)** CD8 T cell viability (left) and suppression ratio (right) for 1:1 CD8: Ly6C+ cells cultured with IgG2a/IgG2b (blue) or 10-0.16 µg αPD-L1/αPDL2 blocking antibodies (yellow).. Data are represented as mean ± SEM.

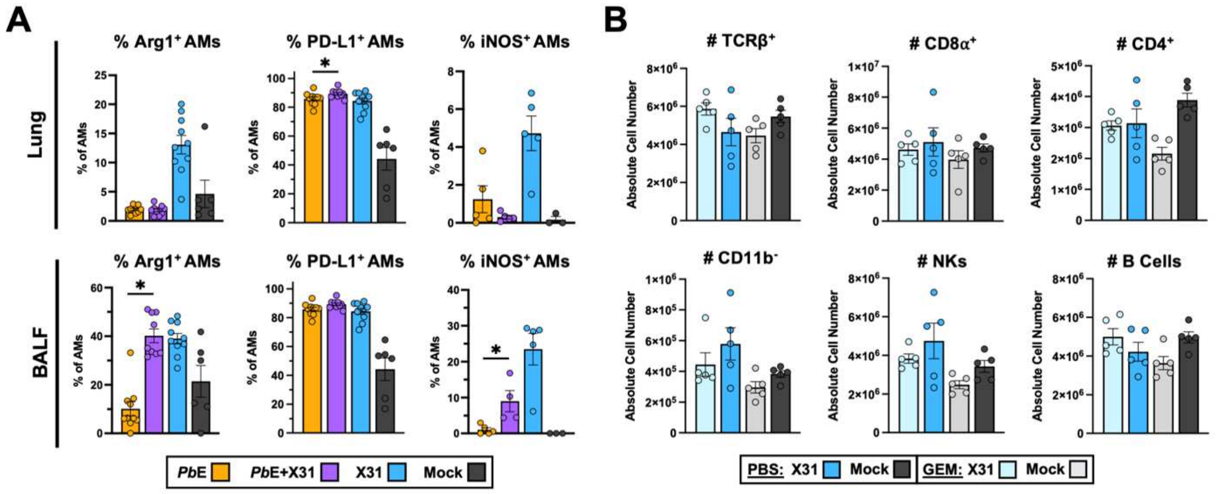

Supplemental Figure 5. At 6 DPI the frequency of PDL1^{+} AMs in the lung (p=0.023) and the frequency of Arg1+ (p=0.0002) and iNOS+ (p=0.016) expressing AMs is greater in the BALF of co-infected animals relative to *Pb*E singly-infected animals (Mann-Whitney U), (n=3-5 mice per group) **(A)**. Gemcitabine depletion of Ly6C+ subsets does not significantly modulate other immune cell types trafficking to the lung in response to X31 infection. At 6 DPI with X31, there was no difference in the number of TCRβ^{+} cells, CD8 T cells, CD4^{+} T cells, CD11b^{-} cells, NK cells, or B cells post-GEM treatment (Mann-Whitney U all P>0.05) **(B)**. Data are represented as mean ± SEM.

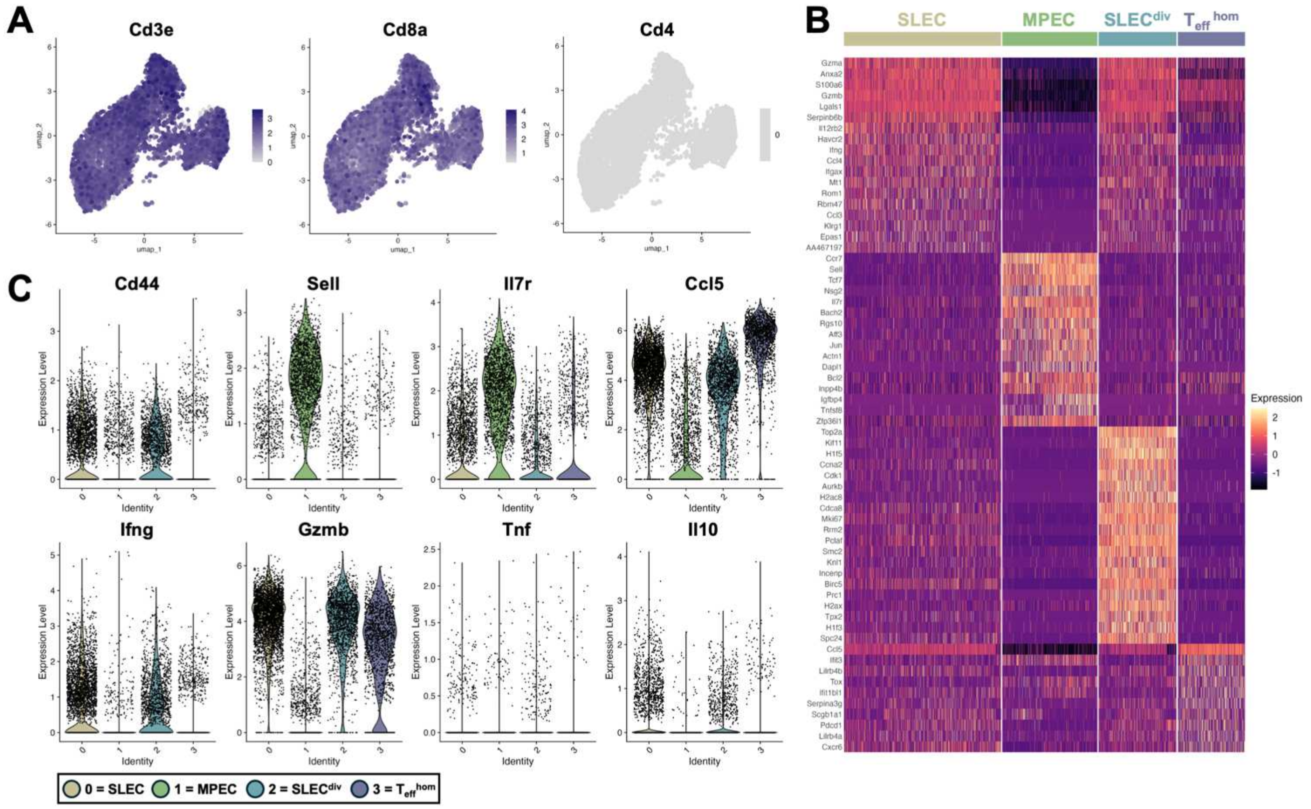

Supplemental Figure 6. Single-cell analysis identified four subsets of CD8 T cells in the lung of co-infected mice. (A) Feature plots to verify sample purity showing high expression of CD3ε (left) and CD8α (center) with no expression of CD4 (right). **(B)** Heat map showing the top 20 expressed genes for each of the four CD8$^+$ T cell clusters, which were used to identify them as SLEC, MPEC, SLEC^{div}, and T_{eff}^{hom}. **(C)** Violin plots showing expression of key genes for all four clusters, further verifying cluster identity. Ccl5: C-C motif chemokine ligand 5; gzmb: granzyme B; IFNg: interferon-γ; IL7r: interleukin receptor; MPEC: memory progenitor effector cells; Sell: CD62L; SLEC: short-lived effector cells; T_{eff}^{hom}: homing T effector cells; TNF: tumor necrosis factor.

Supplementary Information

Supplementary Table 1. Flow cytometry panel to identify and phenotype myeloid cells.

Fluorophore-Marker	Clone	Dilution	Vendor
BUV395–CD4	GK1.5	1:400	BD
BUV496–CD8α	53-6.7	1:400	BD
BUV563–CD80	16-10A1	1:200	BD
BUV615–TCRβ	H57-597	1:200	BD
BUV661–CD19	1D3	1:400	BD
BUV737–F4/80	T45-2342	1:100	BD
BUV805–CD86	PO3	1:100	BD
BV421–CD11c	N418	1:200	BD
ZombieAqua Fixable Viability Dye	-	1:1000	BioLegend
BV570–Ly6C	HK1.4	1:400	Invitrogen
BV650–CD11b	M1/70	1:400	BioLegend
BV711–CD11c	N418	1:400	BioLegend
BV785–Ly6G	1A8	1:400	BioLegend
PerCP–CD45	104	1:200	BD
FITC–I^A/I^E (MHC-II)	M5/114.15.2	1:200	BioLegend
NovaFluor Blue/610-70S–Siglec F	1RNM44N	1:100	Invitrogen
PE–iNOS	W16030C	1:100	BioLegend
PE-Cy7–CD64	X54-5/7.1	1:400	BioLegend
APC–CD161 (NK1.1)	PK136	1:400	BioLegend
Alexa Fluor 700–Arg1	A1exF5	1:100	Invitrogen

Supplementary Table 2. Flow cytometry panel to quantify cytokine production by CD8 T cells.

Fluorophore-Marker	Clone	Dilution	Vendor
BUV496–CD4	GK1.5	1:400	BD
BUV563–KLRG1	2F1	1:100	BD
BUV615–TCRβ	H57-597	1:200	BD
BV421–CD8α	53-6.7	1:400	BioLegend
BV510–CD62L	MEL-14	1:200	BioLegend
BV711–IFN-γ	XMG1.2	1:200	BioLegend
BV785–CD127	A7R34	1:200	BioLegend
FITC–CD44	IM7	1:200	BioLegend
PerCP–CD45.2	104	1:200	BioLegend
PE–IL-10	JES5-16E3	1:200	BD
PE-Cy7–TNF-α	MP6-XT22	1:200	BioLegend
APC–GzmB	QA16A02	1:200	BioLegend
Zombie NIR Fixable Viability Stain	N/A	1:1000	BioLegend

Supplementary Table 3. Flow cytometry panel to sort $CD8^+$ $CD44^+$ T Cells for the 2D-MP assay.

Fluorophore-Marker	Clone	Dilution	Vendor
BV421–CD44	IM7	1:200	BioLegend
FITC–CD4	RM4-5	1:200	BD
PE–TCRβ	H57-597	1:200	Invitrogen
APC–CD8α	53-6.7	1:200	BioLegend
ZombieNIR Fixable Viability Stain	-	1:1000	BioLegend
